# Hospital Contracts and Prices

**Published:** 1910-05-14

**Authors:** 


### Special Departmental Article.

**Contributions and questions on matters affecting this department of an institution are invited.**

### HOSPITAL CONTRACTS AND PRICES.

### V.—SURGICAL INSTRUMENTS, WINES, AND SUNDRIES.

#### Surgical Instruments and Appliances.

Having discussed "Drugs and Dressings," we have now to deal with "Surgical Instruments and Appliances," and this is probably the most unsatisfactory item with which hospital committees have to deal. They can exercise comparatively little control over the expenditure for surgical instruments. They can, of course, say when requisitions for instruments are received from the surgical staff that the institution cannot afford to spend more than a certain amount. They can also suggest that some further service may be exacted from the instruments already in possession; but, after all, there is a distinct feeling on their part that the surgeons who perform the operations are best qualified to judge of the usefulness, or otherwise, of the instruments with which they have to work, and there is a distinct desire to meet their wishes if possible. Occasionally, we are sorry to say, there is a tendency upon the part of some honorary surgeons to throw aside useful instruments, and also to ask to be supplied with fancy instruments, which may never be used, in consequence of which unnecessary expense is incurred; but it is not so much upon the lack of control inside the hospital as the difficulty of obtaining competitive prices on identical lines from instrument makers generally to which we refer when we state that "surgical instruments and appliances" is an unsatisfactory sub-head from a hospital committee's point of view. Instrument makers publish printed catalogues of these goods more or less complete; the prices quoted appear exceedingly high, and there is a uniformity in this respect in those of the best English firms which is exasperating to the economist. Then, again, surgeons have particular ideas. A surgeon must have a particular instrument from a particular firm, and then sometimes the hospital committee discover that it can be obtained from another maker at 10 to 15 per cent. less than from the specially named firm. The instrument from the particular firm may possibly be worth the additional cost, or it may not; it is difficult to judge; and the result is that if the wishes of the surgeon are complied with the economist doubts if there has not been a 10 to 15 per cent. waste in the cost of the article.

We stated just now that the prices in the instrument catalogues always appear high, but when the cost of experiments, advertising, catalogues, etc., is taken into consideration it is not surprising that surgical instruments are costly, and that the firms who specialise in these goods are averse to making reductions from their published prices.

We have discussed the question of the purchase of instruments with keen buyers in different hospitals, and we find that the practice usually followed is to purchase most of the instruments from one instrument maker whose goods have found favour with the honorary surgeons of that particular hospital, occasionally, however, ordering special articles from other firms. We find in a few instances that when instruments are required, two or three firms are asked to submit samples on approval; the samples are compared, and those considered to be the best for the money are finally selected, the rejected instruments being returned. This method is possibly a little troublesome both to the hospital and the manufacturer, but it is fair competition, and the slight variation in pattern between different makers, even if there is no variation in price, gives the surgeons a choice and prevents complaints, as if, after all, they are dissatisfied with their selection they have only themselves to blame. We think that hospitals may do worse than follow this suggestion.

There are, of course, many special appliances included under this sub-head which need special methods of purchase. Electrical and *x*-ray apparatus, theatre and laboratory equipment, etc., which are needed only occasionally, require special knowledge to select, and before any large expenditure upon such goods is embarked upon we strongly recommend the authorities of hospitals contemplating the expenditure to visit other institutions already equipped. There is, fortunately, a species of freemasonry existing between hospital officers. They are proud of their particular institution and will uphold all that is good in it, but when information is required by the authorities of another institution, and advice sought upon any matter of routine or equipment, they will at once give a candid opinion and advise what to avoid and what to adopt. The mutual interchange of visits and of ideas upon hospital methods and equipment is to be strongly commended, and the British Hospitals' Association and the Hospital Officers' Association are organisations which should do much to promote and increase the co-operative spirit between medical charities. Better methods of purchasing, improved methods of administration, keener supervision of contractors and contracts, will assuredly result from a wider interchange of ideas between hospital administrators.

#### Wines and Spirits.

The consumption of wines and spirits is steadily decreasing. The idea which prevailed in the medical profession some years ago that almost all sick people must be placed upon stimulants has died a natural death, and now comparatively few are ordered alcohol. The new spirit duties will, of course, increase the cost per head under this sub-head without an increase in the quantity consumed, and they will also increase the drug bill considerably. There is no doubt that the charitable public will be called upon to find several thousands of pounds annually to defray the additional expenditure upon a great many drugs containing alcohol dispensed from the dispensaries of our great medical charities, and it is to be hoped that the Government may be able to find a method of granting relief to all hospitals, seeing the very great difficulty they have in making their ordinary income meet their ordinary expenditure.

Individual hospitals will, of course, have different methods of purchasing wines and spirits, but an annual contract for such quantities as may be required is a very good arrangement, samples and prices being submitted for selection. Expensive wines and high-priced spirits are not necessary for hospital use. The special flavour for which connoisseurs are prepared to pay a long price is not needed. The wine or spirit is ordered because the patient needs stimulating, and so long as they are good and sound, medium-priced articles will convey to the system of the patient the alcohol for which alone the wine or spirit is ordered.

### Dispensary Sundries.

We have now only to deal with the question of "Dispensary Sundries," *i.e.* bottles, corks, etc. Bottles to be well bought should be purchased in as large quantities as possible, and if a contract covering a period is entered upon it should not be for a shorter period than 12 months. As a rule, bottles have to be specially made, as it is advisable to have the name of the institution upon them; and as it is the usual practice to charge for the preparation of the necessary moulds, it is a distinct advantage to have a lengthy contract, or, as an alternative, order at one time as large a supply of bottles as possible.

In a contract we have recently examined, which runs until December next, we find the following prices for bottles of good quality, which appear to be distinctly favourable:—

| | | Per gross. |
|---|---|---|
| White Flints, Oval, Lettered ... ... | 1 oz. | 8s. 6d. |
| " " " " ... ... | 2 oz. | 10s. 6d. |
| Green Flints, Dispensing, Lettered ... | 4 oz. | 6s. 6d. |
| " " " " ... | 6 oz. | 7s. 6d. |
| " " " " ... | 8 oz. | 7s. 6d. |
| " " " " ... | 12 oz. | 11s. 3d. |
| Actinic Green, Poisons, Lettered ... | 1 dram | 4s. 0d. |
| " " " " ... | 2 dram | 4s. 0d. |
| " " " " ... | 1 oz. | 5s. 9d. |
| " " " " ... | 2 oz. | 6s. 9d. |
| " " " " ... | 4 oz. | 8s. 0d. |
| " " " " ... | 6 oz. | 10s. 6d. |
| " " " " ... | 8 oz. | 10s. 6d. |
| " " " " ... | 12 oz. | 15s. 0d. |
| " " " " ... | 16 oz. | 18s. 0d. |
| " " " " ... | 20 oz. | 21s. 0d. |
| " " " " ... | 40 oz. | 38s. 0d. |
| " " " " ... | 80 oz. | 80s. 0d. |

In some hospitals we find it is the practice to keep a separate bottle account. The sums paid by out-patients for bottles are set aside, and the money thus obtained pays for the whole of the bottles, corks, labels, etc., required. Dispensary labels should, of course, be obtained from the printing firms who specially lay themselves out to supply these goods. If obtained from the ordinary printers they are exceedingly costly.

